# Maternal and Cord Serum Cytokine Changes with Continuous and Intermittent Labor Epidural Analgesia: A Randomized Study

**DOI:** 10.1100/2012/607938

**Published:** 2012-05-01

**Authors:** Venkat R. Mantha, Manuel C. Vallejo, Vimala Ramesh, Bobby L. Jones, Sivam Ramanathan

**Affiliations:** ^1^Department of Anesthesiology, Magee-Womens Hospital of UPMC, University of Pittsburgh Medical Center, Pittsburgh, PA 15213, USA; ^2^Department of Anesthesiology, Passavant Hospital, University of Pittsburgh Medical Center, Pittsburgh, PA 15237, USA; ^3^Department of Psychiatry, Western Psychiatric Institute and Clinic, University of Pittsburgh Medical Center, Pittsburgh, PA 15213, USA; ^4^Department of Anesthesiology, Cedars-Sinai Medical Center, Los Angeles, CA 90048, USA

## Abstract

*Background*. Maternal fever during labor epidural analgesia (LEA) may cause increased maternal and cord serum inflammatory cytokines. We report the effects of intermittent and continuous LEA on these cytokines. *Methods*. Ninety-two women were randomly assigned to continuous (CLEA) or intermittent (ILEA) groups, 46 in each. Maternal temperature was checked and blood drawn at epidural insertion (baseline) and four-hourly until 4 h postpartum (4 PP). Cord blood was drawn after placental delivery. Interleukin-1**β** (IL-1**β**), interleukin-6 (IL-6), interleukin-8 (IL-8), granulocyte macrophage-colony stimulating factor (GM-CSF), and tumor necrosis factor-**α** (TNF-**α**) were measured and analyzed according to group randomization, and then combined and reanalyzed as febrile (temperature ≥38°C) or afebrile groups. *Results*. Significant intragroup changes from baseline were noted in some groups. Data are pg/mL, median (Q1/Q3). IL-6 rose at all time points in all groups. *CLEA*: baseline: 18.5 (12.5/31.1), 4 h: 80.0 (46.3/110.8), 8 h: 171.9 (145.3/234.3), and 4 PP: 81 (55.7/137.4). *ILEA*: baseline: 15.7 (10.2/27.1), 4 h: 68.2 (33.3/95.0), 8 h: 125.0 (86.3/195.0), and 4 PP: 70.2 (54.8/103.6). *Febrile group*: baseline: 21.6 (13.8/40.9), 4 h: 83.9 (47.5/120.8), 8 h: 186.7 (149.6/349.9), and 4 PP: 105.8 (65.7/158.8). *Afebrile group*: baseline: 10.9 (2.1/17.4), 4 h: 38.2 (15.0/68.2), 8 h: 93.8 (57.1/135.7), and 4 PP: 52.9 (25.1/78). IL-8 rose at all time points in *CLEA*: baseline: 2.68 (0.0/4.3), 4 h: 3.7 (0.0/6.5), 8 h: 6.0 (3.3/9.6), 4 PP: 5.6 (0.8/8.0), and *afebrile group* baseline: 2.5 (0.0/4.7), 4 h: 3.3 (0.0/6.2), 8 h: 5.3 (1.9/9.8), and 4 PP: 4.7 (0.0/7.6). It fell at 4 PP in *febrile group*: baseline: 4.1 (0.0/6.4), 4 h: 3.8 (0.0/6.5), 8 h: 5.2 (2.5/8.0), and 4 PP: 2.9 (0.0/4.0). GM-CSF increased at 8 h and decreased at 4 PP in *ILEA* baseline: 2.73 (0.0/7.2), 4 h: 2.73 (0.0/7.9), 8 h: 3.9 (2.7/11.5), and 4 PP: 2.0 (0.0/7.2). It increased at 4 h and 8 h and decreased at 4 PP in *febrile* group: baseline: 2.6 (0.0/4.2), 4 h: 3.2 (2.1/7.0), 8 h: 4.0 (3.2/12.3), and 4 PP: 2.4 (1.7/12.6). There were no intergroup cytokine changes in maternal or cord serum in CLEA versus ILEA or febrile versus afebrile groups. *Conclusions*. Some cytokines, especially IL-6, rise physiologically during labor epidural analgesia.

## 1. Introduction

Maternal intrapartum fever may occur with labor epidural analgesia (LEA)[1]. The mechanism(s) of the fever are not fully understood, but may result from disturbances in thermoregulatory mechanisms [1, 2]. However, there are reports that fever may represent a maternal inflammatory response. Goetzl et al. [3] and Smulian et al. [4] reported a significantly increased maternal serum interleukin-6 (IL-6) levels in febrile versus afebrile subjects.

We previously reported that intermittent, versus continuous, LEA had a lower incidence of maternal and neonatal fever in the first few hours of epidural analgesia initiation [5]. In the same study, we collected, but did not fully analyze, maternal and cord serum cytokine levels; we now report those results. To our knowledge, there are no studies comparing maternal serum cytokine changes with continuous versus intermittent epidural analgesia.

## 2. Methods

After Institutional Review Board (IRB) approval, 92 women gave informed consent and were enrolled in this prospective, randomized unblinded study. As required by our IRB, a parent or legal guardian provided consent for enrolled minors. A computer-generated program (Quattro Pro; Corel Corp., Ottawa, Canada) randomized an equal number of women (46) to receive continuous or intermittent lumbar epidural analgesia (CLEA and ILEA groups, resp.). Inclusion criteria were a healthy, nulliparous parturient in spontaneous labor with a term, singleton fetus in vertex presentation. Exclusion criteria were a baseline temperature of ≥37.5°C, suspected chorioamnionitis, history of drug abuse, and pregnancy-related complications such as preeclampsia, intrauterine growth retardation, and gestational diabetes mellitus.

One of the investigators initiated the epidural analgesia at maternal request, using strict sterile precautions (i.e., sterile gloves, shoe covers, operating room scrub, cap, and facemask). In all patients, LEA was initiated with a 3 mL test dose of 1.5% lidocaine with 1 : 200,000 epinephrine, followed by fentanyl 100 *μ*g and 8 mL of an analgesic solution: either 0.125% bupivacaine with fentanyl 0.0002% or 0.1% ropivacaine with fentanyl 0.0002%. In the CLEA group, a continuous infusion of one of the analgesic solutions was administered at 10–15 mL/hr with a goal of maintaining a T-10 or higher sensory blockade. In the ILEA group, 10–15 mL of the solution was given by manual injections as requested by the patient. The labor and delivery rooms were maintained at a temperature comfortable for the patient, generally between 20 and 22°C.

### 2.1. Data Collection

 Baseline measurements noted at initiation of epidural analgesia included maternal age, height, weight, gestational age, cervical dilatation, and tympanic temperature. Peripheral venous blood (5 to 10 mL) was also drawn at this time for cytokine analysis. The temperature measurement and venipuncture were repeated at four-hourly intervals, with the final sampling occurring four hours postpartum (4 PP). Following fetal and placental delivery, umbilical vein cord blood was collected for cytokine analysis. A pediatric nurse measured the neonatal rectal temperature within 30 minutes of delivery.

The blood samples were collected in serum separation tubes and refrigerated immediately. Within 24 hours, the serum was separated by centrifugation at 2500 g for 20 minutes (Sorvall Biofuge Primo, Thermo Fisher Scientific, Ashville, NC, USA). The serum was then placed in duplicate aliquots and immediately frozen and stored at −70°C until analysis. The samples were batched in groups of 50–90 samples for laboratory analysis. They were analyzed within 3 months of collection, by a Multiplex Bead Immunoassay technique (BioSource International Inc., Camarillo, CA, USA). The serum cytokine levels assayed were interleukin-1*β* (IL-1*β*), interleukin-6 (IL-6), interleukin-8 (IL-8), granulocyte macrophage colony-stimulating factor (GM-CSF), and tumor necrosis factor-*α* (TNF-*α*). The sensitivity of the test for IL-1*β*, IL-6, IL-8, GM-CSF, and TNF-*α* was 15 pg/mL, 3 pg/mL, 3 pg/mL, 15 pg/mL, and 10 pg/mL, respectively. The respective intra- and inter-assay precision (in pg/mL, mean (SD)) for the different cytokines was: IL-1*β*: 1410 (117) and 1440 (127), IL-6: 883 (58) and 892 (62), IL-8: 1020 (73) and 987 (97), TNF-*α*: 1130 (92) and 1130 (94), and GM-CSF: 2137 (75) and 2084 (98).

Labor and epidural characteristics like the number of vaginal examinations, use of internal monitors or oxytocin augmentation, clinical suspicion of chorioamnionitis, group *B* streptococcal colonization of the genital tract, times of membrane rupture and full cervical dilatation, time and type of delivery, epidural insertion to full dilatation and delivery, and neonatal sepsis evaluation rate were collected. At our institution, clinical chorioamnionitis is diagnosed on the basis of maternal fever (temperature ≥38°C) plus one or more of the following: maternal or fetal tachycardia, uterine tenderness, foul smelling amniotic fluid, or maternal leukocytosis [5]. Clinical suspicion of chorioamnionitis is treated aggressively with intravenous antibiotics and rectal acetaminophen every 4–6 hours as needed. Placentas were sent for histological examination at the discretion of the obstetricians. Guidelines for neonatal sepsis evaluation (NSE) at our institution include prolonged rupture of membranes (>18 hours) and chorioamnionitis; neonatal conditions include hypo- or hyperthermia at 6 hours after birth, apneic spells, respiratory distress or pneumonia, central cyanosis, listlessness, convulsions, poor feeding or vomiting, and cardiovascular instability. NSE at our institution is done at the discretion of the neonatologists.

### 2.2. Data Analysis

A primary analysis between the two groups was performed. The two groups were then combined, and a reanalysis made between “febrile” and “afebrile” groups to look for any association between cytokines and the development of fever. Parturients who developed intrapartum fever (temperature ≥38°C) were classified as the febrile group and the rest as the afebrile group.

Demographic data were analyzed by Student's *t-*test. Distributions of cytokine levels were markedly skewed; thus nonparametric methods were used for the cytokine investigations. Tests of changes in cytokine levels over time, and comparisons of those changes by infusion method and presence of fever were analyzed using the Wilcoxon sign and sign-rank tests. For statistical significance, a more stringent threshold of *P* < 0.01 was used to control for multiple comparisons. This was based on the false discovery rate method [6].

The sample size was determined for our original study based on the assumed incidence of fever with CLEA and ILEA [5].

## 3. Results

No technical problems or complications were encountered during the insertion or maintenance of epidural analgesia. There were no differences between the CLEA and ILEA groups in demographic and baseline data (age, height, weight, gestational age, cervical dilatation, and temperature) (Table 1).

### 3.1. Maternal Temperature 

Details of the maternal temperature changes with CLEA and ILEA have been previously reported [5]. Briefly, the incidence of fever in ILEA at 4 h was lower than in CLEA (2/42, 4.6% versus 10/44, 22.7%, *P* = 0.036). At 8 h and thereafter, no differences were noted. The overall incidence of fever was 14/46 (30.4%) in CLEA and 12/43 (27.9%) in ILEA (*P* = *n*s). Regrouping based on the development of fever resulted in 26 women falling into the febrile group and 66 into the afebrile group. Highly significant differences were seen between these two groups at all time points (Table 2).

### 3.2. Neonatal Temperature

As shown in Table 2, neonates born to febrile mothers had significantly higher temperatures. Two neonates, both in the afebrile group, underwent NSE, which was negative.

### 3.3. Maternal Serum Cytokines

**Figure 1:**
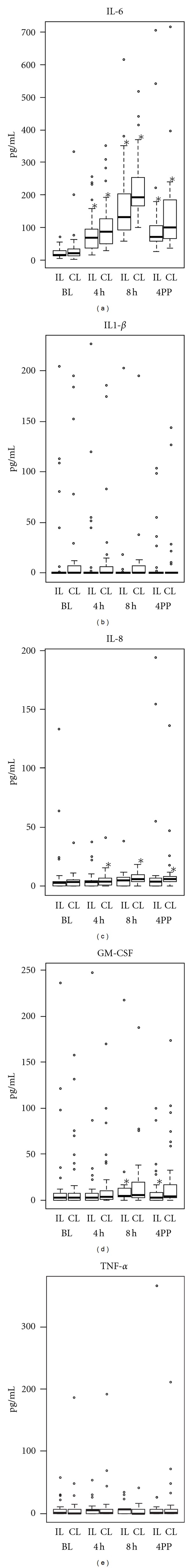
Box plots of intra- and intergroup maternal serum cytokine changes by infusion method during labor epidural analgesia. IL-6: interleukin-6, IL-1*β*: interleukin-1*β*, IL-8: interleukin-8, GM-CSF: granulocyte macrophage-colony stimulating factor, TNF-*α*: tumor necrosis factor-*α*. IL: ILEA group. CL: CLEA group. BL: baseline. 4 h and 8 h: 4 and 8 hours from baseline, respectively. 4 PP: 4 hours postpartum. *: Significant intragroup changes from baseline. There were no intergroup differences.

**Figure 2:**
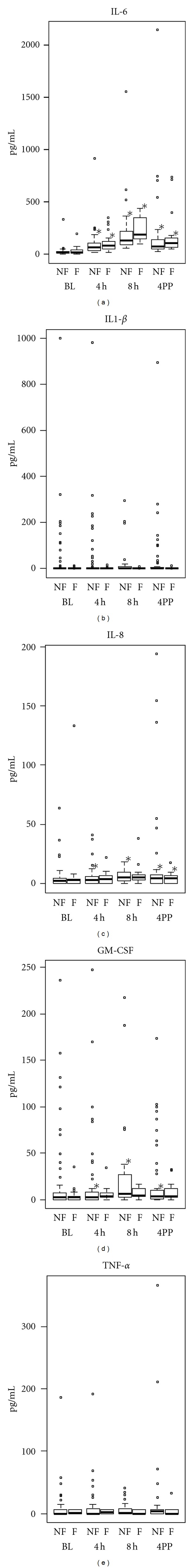
Box plots of intra- and intergroup maternal serum cytokine changes by fever status during labor epidural analgesia. IL-6: interleukin-6, IL-1*β*: interleukin-1*β*, IL-8: interleukin-8, GM-CSF: granulocyte macrophage-colony stimulating factor, TNF-*α*: tumor necrosis factor-*α*. NF: afebrile group. F: febrile group. BL: baseline. 4 h and 8 h: 4 and 8 hours from baseline, respectively. 4 PP: 4 hours postpartum. *: Significant intragroup changes from baseline. There were no intergroup differences.

Intragroup and intergroup analysis of changes from baseline is shown by infusion type in Figure 1 and by fever status in Figure 2. Intragroup analysis showed the following changes: IL-6 showed a highly significant rise (*P* ≤ 0.0001) at all time points in all groups—CLEA, ILEA, febrile and afebrile. IL-8 also showed a highly significant rise (*P* ≤ 0.0075) at all time points in CLEA and afebrile groups; in the febrile group, it showed a fall at 4 PP, while in ILEA there was no change.GM-CSF increased at 8 h and decreased at 4 PP in ILEA; in the afebrile group, it increased at 4 h and 8 h and decreased at 4 PP, while in CLEA and febrile groups there was no change. There was no change in IL-1*β* or TNF-*α* at any time point in any group. Intergroup analysis of CLEA versus ILEA and febrile versus afebrile groups showed no significant difference in any cytokine at any time point.

### 3.4. Cord Serum Cytokines

**Figure 3:**
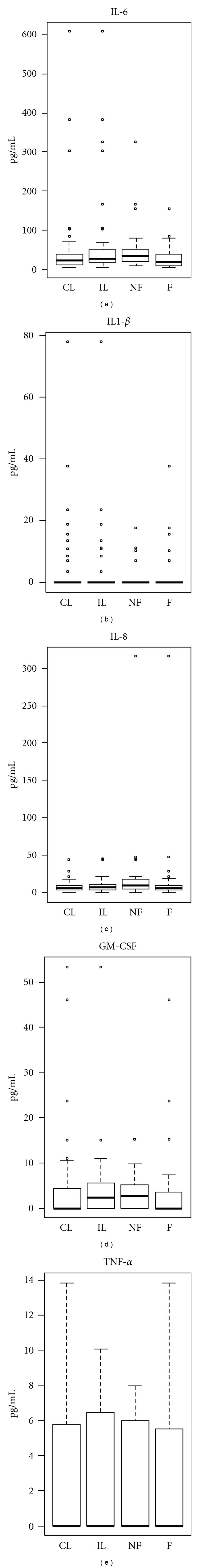
Box plots of cord serum cytokines in the four groups studied. IL-6: interleukin-6, IL-1*β*: interleukin-1*β*, IL-8: interleukin-8, GM-CSF: granulocyte macrophage-colony stimulating factor, TNF-*α*: tumor necrosis factor-*α*. IL:ILEA group. CL: CLEA group. NF: afebrile group. F: febrile group. There were no significant CLEA versus ILEA or F versus NF differences in any cytokine.

There were no significant differences between CLEA and ILEA. Although neonates born to febrile versus afebrile mothers had higher temperatures (Table 2), there were no differences between these groups in any cytokine (Figure 3).

### 3.5. Labor and Epidural Characteristics

The labor and epidural characteristics of CLEA and ILEA were shown in our previous publication [5], and those in febrile and afebrile subjects are shown in Table 3. Briefly, febrile subjects had significantly lower baseline cervical dilatation, more vaginal examinations and use of internal monitors, longer time intervals between epidural insertion to full dilatation, epidural insertion to delivery, rupture of membranes to full dilatation, and rupture of membranes to delivery.

## 4. Discussion

To our knowledge, the effect of intermittent versus continuous LEA on maternal and cord serum cytokine expression has not been previously reported.

Our data indicate that some maternal serum cytokines increase with LEA. IL-6 exhibited the most consistent intragroup change, increasing during labor and remaining elevated for at least 4 h postpartum, regardless of CLEA or ILEA and febrile or afebrile group status. There were no CLEA/ILEA or febrile/afebrile intergroup differences in maternal or cord sera. Thus, it is interesting to note that at the 4 h time point, a significantly higher incidence of fever in CLEA versus ILEA group yielded no differences in IL-6 levels. Thus, maternal and cord IL-6 levels appear independent of the method of epidural analgesia, the development of fever, and neonatal temperature.

Our findings differ from some studies that have evaluated maternal serum IL-6 levels and intrapartum fever. Goetzl et al. [3], whose patients received continuous epidural infusions, found that IL-6 levels increased at 4 and 8 h following epidural analgesia initiation. Smulian et al. [4], in a case-control, mixed parity study, found that maternal IL-6 was a strong marker for intrapartum fever; however, only one blood sample per subject was collected within an hour of delivery, and not all subjects received epidural analgesia. De Jongh et al. [7] studied a group of mixed parity patients to evaluate the influence of analgesia (with or without epidural) or anesthesia (general versus epidural) technique and type of delivery (vaginal or cesarean) on peripartum maternal serum IL-6 levels. They found that serum IL-6 levels were significantly higher in parturients who had epidural analgesia; however, the rate of fever, if any, in these subjects was not mentioned.

Rise of maternal serum cytokines, particularly IL-6 may be a normal response to labor [8–13]. Arntzen et al. [8] found that the strength and frequency of uterine contractions were significantly related to serum IL-6 levels. Austgulen et al. [9] observed that IL-6 increased with gestational age and labor activity. Buonocore et al. [10] found that during normal vaginal delivery, IL-6 levels were higher during labor and immediately after delivery than at the beginning of labor. According to Hebisch et al., IL-6 (also IL-8) levels rose with labor onset and remained high through postpartum day 3. Opsjln et al. observed that maternal serum IL-6 may plays a role in the onset of normal labor. Unfortunately, these studies do not mention maternal fever status or use of epidural analgesia.

Serum IL-6 levels also physiologically increase in response to high-intensity exercise in humans [14–19], which may include labor associated frequent, high-intensity uterine contractions and psychological stresses [7]. IL-6 is a pleiotropic cytokine that has multiple effects on the host including the ability to induce fever. As a potent inducer of acute phase responses, it may have a protective effect on the host [20]. Childbirth can induce an acute phase response [21].

We therefore do not think that a rise in IL-6 represents a pathological response. Our study certainly found that to be true.

We found statistically significant intragroup changes in other cytokines. Clinical significance of these findings is unclear. Our findings have similarities and differences from other studies in the obstetric population. In the Goetzl et al. [3] study, IL-8 levels insignificantly rose at 4 and 8 hours compared to baseline. IL-1 (or 1*β*) was either undetected [8] or no change found during labor [3, 9, 13], while it was found to be elevated in two other studies [10, 12]. The majority of the studies did not find increases in TNF-*α* or GM-CSF during labor.

In contrast to our study, Smulian et al. [4]and Goetzl et al. [3] found significantly higher cord blood IL-6 levels in febrile parturients. Goetzl et al. [3] attributed “fetal inflammation” as being caused by maternal inflammation, with greater maternal than cord IL-6 levels. It is unclear whether maternal or cord blood cytokines are interrelated. Although De Jongh et al. [22] found that IL-6 levels in the cord appeared dependent on maternal levels, a number of reports suggest that cytokines, including IL-6, do not cross the term placenta [23, 24].

Limitations in our study include the use of different local anesthetics (ropivacaine and bupivacaine), which may have confounded our results. However, as discussed in our paper that studied the same patients [5], we found little or no difference between these two solutions in VAS pain scores, local anesthetic usage, and duration of labor or mode of delivery.

Another limitation is that we did not have a control group that did not receive epidural analgesia. Such a study would be difficult at our institution, where approximately 90% of laboring women receive epidural analgesia.

In conclusion, we found that maternal serum inflammatory cytokines rise during LEA, irrespective of the method of epidural dosing (intermittent or continuous) or the development of fever. These findings suggest that a rise in maternal serum cytokines during LEA may be physiological, and that there may be no pathological basis for epidural fever based on proinflammatory cytokine analysis.

## Figures and Tables

**Table 1:** Demographic data.

	CLEA	ILEA	*P*
	*n* = 46	*n* = 46	
Age	27.0 ± 7.2	26.5 ± 6.5	0.74
Height (cm)	160.4 ± 6.1	160.8 ± 7.1	0.77
Weight (kg)	80.1 ± 17.8	81.3 ± 16.7	0.73
Gestational age (weeks)	39.8 ± 1.1	39.4 ± 1.3	0.12
Baseline cervical dilatation	3.4 ± 1.5	3.5 ± 1.5	0.83
Baseline temperature (°C)	36.7 ± 0.6	36.6 ± 0.5	0.49

Data are mean ± standard deviation.

**Table 2:** Maternal and neonatal temperature changes in febrile and afebrile groups.

Variable	Febrile °C	*n*	Afebrile °C	*n*	*P*
Baseline	36.9 ± 0.44	26	36.5 ± 0.55	63	0.003
4-hour	37.7 ± 0.52	26	37.0 ± 0.58	57	<0.001
8-hour	38.3 ± 0.40	20	37.3 ± 0.48	24	<0.001
4-hour PP	37.6 ± 0.52	26	37.0 ± 0.56	63	<0.001
Neonatal	37.0 ± 0.44	26	36.7 ± 0.50	63	0.012

Data are mean ± standard deviation. 4-hour PP = 4-hour postpartum.

**Table 3:** Labor and epidural characteristics of febrile and afebrile subjects.

	Febrile	*n*	Afebrile	*n*	*P *
Baseline cervical dilatation (cm)	2.8 ± 1.7	26	3.7 ± 1.4	66	0.001
Number of vaginal examinations	6.6 ± 2.5	26	5.5 ± 2.0	63	0.04
Internal monitors	54%	14/26	29%	18/63	0.04
AROM	69%	18/26	62%	39/63	0.68
Oxytocin augmentation	81%	21/26	73%	46/63	0.62
Chorioamnionitis	27%	7/26	6%	4/63	0.15
GBS positive	19%	5/26	17%	11/66	1.0
Assisted/operative delivery	54%	14/26	49%	31/63	0.86
ROM to full dilatation (min)	479 ± 288	23	347 ± 244	54	0.04
ROM to delivery	605 ± 300	26	467 ± 284	63	0.04
Full dilatation to delivery (min)	120 ± 80	23	100 ± 84	56	0.33
Epid. insert. to full dilatation (min)	357 ± 130	23	270 ± 168	56	0.03
Epid. insert. to delivery (min)	481 ± 141	26	380 ± 204	63	0.02

Data are mean ± standard deviation or number.

AROM: artificial rupture of membranes; ROM: rupture of membranes; GBS positive: group *B* streptococcal colonization of genital tract; Assisted/operative delivery: forceps, vacuum, or cesarean section; Epid. insert.: epidural insertion.
